# Supplementary material for: Multicenter study of pneumococcal carriage in children 2 to 4 years of age in the winter seasons of 2017-2019 in Irbid and Madaba governorates of Jordan
Source: PLoS One. 2020 Aug 13;15(8):e0237247. doi: 10.1371/journal.pone.0237247 (PMC7425857; doi:10.1371/journal.pone.0237247)
Supplement: Attachment — Submitted filename: Response to reviewers.docx [file pone.0237247.s007.docx]

**Response to reviewers**

Thank you for the valid feedback received through the mail on 3^rd^ of April 2020. Below, please find the response to the reviewers’ comments.

| **Response to reviewer 1** | | |
| --- | --- | --- |
|  | Comments | Response |
| Major issue | A major concern is the confusing nature of how the results presented. Many of the finer details can be summarized in the text referring to charts for support, highlight interesting differences for the reader to note. The numerous tables presenting the finer details can be presented in supplementary materials. | Some representative tables were changed into charts and figures and that their detailed tables were provided as supplementary material as requested |
| Minor issues |  |  |
| Page 2, line 35 | “DCCs” should be defined on line 27 | DCCs was defined in line 27 as first time mentioned in the text |
| Page 2, line 41 | “PCV13 “ should be defined | Was changed to “the thirteen valent pneumococcal conjugate vaccine (PCV13)” |
| Page 2, line 41 | PCV13 serotype coverage for Irbid was 65.6%, and for all regions of Madaba 65.3%.” Since the rates are pretty much the same, for the abstract I suggest something similar to “Serotype coverage of the 13 valent pneumococcal vaccine (PCV13) was about 65% for both regions.” | Was done or changed as suggested to: Serotype coverage of the thirteen valent pneumococcal conjugate vaccine (PCV13) was about 65% for both regions |
| Page 2, line 42 | “All vaccine serotypes isolated in this study included in the PCV13 showed penicillin resistance from 96.4% to 100% with exception to serotypes 3 and 5 in both cities.” Suggest re-wording to: “Over 96% of isolates with PCV13 serotypes in this study were resistant to penicillin with the exception of serotypes 3 and 5.” | Was done or changed in the abstract as suggested to:  Over 96% of isolates with PCV13 serotypes in this study were resistant to penicillin with the exception of serotypes 3 and 5. |
| Page 3, line 49 | “… (S. pneumoniae)…” is not required here | Done (deleted) |
| Page 6, line 118 | Describe what the level of significance was, ie p<0.05 using 2-tailed values | Done as requested to:  Student t-test was considered for significant differences using 2-tailed values with the level of significance at p<0.05. |
| Page 7, lines 135-138 | If the differences between the two cities are not analysed, the manuscript can be shortened by combining these data for both cities. Keep these details in table 1. Perhaps make table 1 a supplementary table? | Table 1 was changed according to the data available in lines 135-138, and Table 1 was changed to Table 1a with detailed number of NP-samples from each center and was put as supplementary table |
| Page 8 onward | page number missing from draft manuscript | Page numbers were added |
| Line 151 | Suggest using a chart to display this information and put Table 2 into supplementary material. | A chart as Fig 1. was made to display table 2, and table 2 was put as supplementary material as suggested |
| Line 165 | Table 4 not included in draft. Again, maybe use a chart for this information instead of a table? | Table 4 was inserted as Fig 2. As the Table 4 was put as supplementary to the figure |
| Line 171 | If there is no statistical difference, just state as such and combine data for both cities. State detailed data only for the significant differences. The P<0.05 indicates significance? This should be p>0.05? | This is true, was changed accordingly |
| Line 172 | I would suggest separating the monthly coverage variations of coverage from that of the antimicrobial resistance data. Keep the same data type together (ie. 1) monthly/seasonal trend of coverage; 2) AMR distributions geographically/temporal). Furthermore, perhaps the monthly trend of AMR is not needed, but present the just annual /regional differences if present? | Done as suggested, so that in table 5 was only for the coverage rates on monthly basis for both cities, and in table 6 was for the resistance rates for both cities on monthly basis (Annual rates was shown in other table. Figure 3 and 4 were set for these data and tables 5 and 6 will be as supplementary to show the difference between the two cities |
| Lines 200-203 | Data in Tables 8-10 can be summarized to show the AMR results into a chart instead of the data from tables 6-7? Put detailed counts into supplementary material. | Tables 8-10 were summarized in one table to avoid repetition of data. Figure 5 was made and the table was set as supplementary as requested |
| Line 221-223 | Again, for easier reading, if there is no large or interesting differences, just make a general statement with the details. | Done as requested |
| Lines 232-240 | Tables 8-10 seem to repeating data covered in previous tables. It is getting confusing as to what differences the read should be noting | Tables were restructured, so that no repeated data are available anymore |
| Lines 232-240 | Are tables 10-11 necessary? Can the data summarized and combined with previous data tables in a more efficient manner? | Data for tables 8 and 9 were partly fused into table 10 to avoid repetition of data. Table 10 was then changed into chart and the table was left as supplementary material |
| Lines 254- 278 | Tables 11 – 15 should be summarized into a figure if possible | For these tables again to avoid repetition of data, tables 11 and 13 were put in one table, table 12 was deleted, and table 14 and 15 were put in one table. The number of the serotypes and data are huge, so that they could not fit in a chart, hoping you will be ok with this solution |
| Line 283 | Spelling error: “… studding…” ; “studying” ? | Spelling error corrected “Studying” |
|  |  |  |
|  |  |  |
| **Response to reviewer 2** | | |
| Major aspects | Comments | Response |
| Point 1 | The use of English must greatly improve if the paper is to be published in PLOS ONE. The author needs to take inputs from similar papers in order to improve the overall presentation of his work. | The English was improved by a native speaker from England, and more input was inserted from similar papers |
| Point 2 | The “**Introduction**” section of the manuscript requires extensive revision. First, the author needs **to expand the review of literature** that is relevant to his study. Second, **the aim** of the study **needs to be properly highlighted** **and justified.** Instead of setting aim in the frame of a simplistic question (reviewer’s personal point of view), I would **suggest that the author should attempt to present the key objectives of his study with regards to what is currently known (i.e. literature), thus highlighting the added value of the paper.** | The introduction was extended with more literature. Regarding the aims of the study were highlighted at the end of the introduction. Since the study group are well known to be in other countries with the highest carriage, and that the DCCs chosen are all in rural areas visited from families mostly with low income and cannot afford the cost of vaccination with the available PCVs. An important issue here is to show the resulted serotypes with more rates belonging to the PCVs harbouring high rates of resistance |
| Point 3 | **The inclusion criteria for the children must be clearly stated** (for e.g., age group, previous vaccination history considerations and equal population from both the genders). | The age group for this study is 2-4 years was chosen by the funding agency, since it is well known worldwide with high carriage rate and according to the world population review reports, this age group count 673.8 thousand or almost 5% of the total population. All children of this study did not have any previous history of PCV vaccination, and this was included in the text. |
| Point 4 | **Details of the demographic surveillance** site with independent survey information (**Socio-demographic characteristics) should be clearly described**. | Regarding demographic surveillance and the socio-economic characteristics. Age group is fixed in the study. Children belong to families with low to middle income and all DCCs are in rural areas |
| Point 5 | There is no mention of the **sampling procedures followed or details of the sample size calculation** for this study. | Regarding sample size. All possible children of age 2-4 years visiting the chosen DCCs in the period of the study were subject to give NP-swabs. Number of NP-swabs was supposed to be 700 but was increased to 1019 at the end of the study to involve most children of this age group in this study |
| Point 6 | **To my point of view, materials and method section of the manuscript is written in an amateurish way and does not match the quality standards for being publish in PLOS ONE**. The description of the data collection, transportation, laboratory analysis, variable definition and data management & analysis is almost chaotic, while the use of terminology and language is too simplified. **Probably a detail flowchart of methodology including the experiment results should be mentioned. The protocol for sampling from 1019 to 341 strain should be elaborated**. | The section Materials and Methods was completely restructured to meet the standards of PLOS ONE as requested |
| Point 7 | To my view, this is not a proper presentation of the statistical results for a survey study. Again, information provided is very hard to follow. **The author should consult similar papers to view how the data of a huge sampling study over 2 years should be properly presented and justified.** For example, the data given in the manuscript are mainly in tabular form but can be more informative, if represented in the form of graphs or charts. Also, the result could be segmented in different sub-sections emphasizing on characteristics of the population, resistance and carriage rate and serotype distribution. | Most of tabular results were changed into charts or figures for better understanding and that repeated results were deleted. Most figures were included in the manuscript and some of the tables were left as supplementary for proper and more informative presentation. Segmentation of the results is also done for each characteristic alone |
| Point 8 | It is surprising that the paper is owned by only one author, since an extensive work is performed and more than one centers are involved. I wonder how can we acknowledge others involved in the study. | All participants in this study project have received monthly salaries for sample collection and transfer, and have no impact in writing or data analysis in the paper. Furthermore, Only one paediatric in Jordan have published on pneumococci in addition to me. Another point is that my experience at the National Reference Center for Streptococci in Germany has enabled me to establish a new lab at the German Jordanian University and do most of the work independently. |
