# Supplementary material for: Multicenter study of pneumococcal carriage in children 2 to 4 years of age in the winter seasons of 2017-2019 in Irbid and Madaba governorates of Jordan
Source: PLoS One. 2020 Aug 13;15(8):e0237247. doi: 10.1371/journal.pone.0237247 (PMC7425857; doi:10.1371/journal.pone.0237247)
Supplement: Attachment — Submitted filename: Response to reviewers.docx [file pone.0237247.s008.docx]

**Response to reviewers**

Thank you for the valid feedback received on Monday 15^th^ of June 2020. Below, please find the responses to the reviewers’ comments.

| **Response to reviewer 1** | | |
| --- | --- | --- |
|  | Comments | Response |
| Minor issues: | | |
| Lines 138-144: | Some antibiotics are capitalized which should be changed to lower case. | Capitalized antibiotics were changed into small letters as requested |
| Major issues: | | |
| Line 176 - Figure 1: | There seems to be two series of data on the x-axis labelled the same: The first two series “%male carriers 17-18” and “%female carriers 17-18” are labelled the same as series 5 and 6. | Figures 1 and 2 were removed as requested in the last point and kept the tables as supplementary material |
| Line 185 : | Remove table 2. | Removed |
| Figure 3: | Remove columns for % PCV10 and %PCV7. Keep only %PCV13 and % Carriage | Data for PCV7 and PCV10 were removed and kept only % carriage and % PCV13 coverage as requested |
| Figure 4: | Should use x-axis as the temporal variable. | Done as requested |
| Keep Figures 5 and 6, remove figures 1 and 2. | Keep Figures 5 and 6, remove figures 1 and 2. | Figures 1 and 2 were removed as requested and kept Figures 5 and 6. The manuscript text was changed accordingly and kept only the supplementary tables for these figures |
| **Response to reviewer 2** | This paper experimentally demonstrates that a resistance and carriage rates among the age group 2 to 4 years for a period of two years, reached an alarming rate especially among vaccine types and propose that it can be controlled by pneumococcal conjugate vaccination strategies. The authors have clarified almost all of the questions raised in the previous review. However, the resolutions of the graphs provided could be improved. Overall, this version of manuscript has been sufficiently improved. | Much appreciated for the response. Better resolution of the figures was done as requested |
